# Tensile Deformation and Fracture Behavior of Nickel-Based Superalloy DZ951G

**DOI:** 10.3390/ma14092250

**Published:** 2021-04-27

**Authors:** Chenhao Guo, Jinjiang Yu, Jinlai Liu, Xiaofeng Sun, Yizhou Zhou

**Affiliations:** 1 Shi-Changxu Innovation Center for Advanced Materials, Institute of Metal Research, Chinese Academy of Sciences, Shenyang 110016, China; chguo18s@imr.ac.cn (C.G.); jlliu@imr.ac.cn (J.L.); xfsun@imr.ac.cn (X.S.); yzzhou@imr.ac.cn (Y.Z.); 2 School of Materials Science and Engineering, University of Science and Technology of China, Shenyang 110016, China

**Keywords:** DZ951G superalloy, tensile properties, stacking faults, microstructures, deformation mechanisms

## Abstract

DZ951G is a novel developed nickel-based directional solidified superalloy with an incipient high melting point and low density. Compared with DZ417G superalloy, DZ951G superalloy has a higher ultimate tensile strength. At intermediate temperatures, the plasticity and strength were both markedly improved, and an obviously anomalous yield behavior could be observed where the yield strength reached its maximum at 760 °C. Below 600 °C, two competitive modes of dislocations shearing γ′ particles existed, in which one was the formation of stacking faults and another was a/2<101> dislocations shearing. At intermediate temperatures, a transitional phase between shearing γ′ particles and bypassing appeared, and the fracture translated from brittle fracture into ductile fracture. Exceeding 900 °C, bypassing of dislocations was operated under thermal activation. Moreover, short continuous stacking faults still existed at 760 °C. Finally, the various dislocation configurations were rationally illuminated and explained with the intrinsic connection of mechanical properties.

## 1. Introduction

Nickel-based superalloys are extensively applied in the aerospace industry, in marine and industry gas engines, especially in turbine blades, as the core of generating power, due to their excellent comprehensive performance in restraining degradation of mechanical properties and structure stability [1,2], which is attributed to their unique two-phase microstructures. In Ni-based superalloys, γ′ particles play a crucial role in the creep property [3,4], and with different atoms in superalloys [5,6], the mismatch between γ and γ′ phases leads to strengthening [7,8,9,10].

DZ951 superalloy has a high incipient melting temperature and good mechanical properties with a much lower cost [11,12,13]. Recently, DZ951 superalloy has been successfully commercialized as tiles resisting heat gas in combustor and hollow guide vans adjusting gas flowing orientation. However, compared with DZ417G superalloy, its plasticity and transverse mechanical properties at intermediate temperatures still have room for improvement [11]. In this research, the composition of DZ951 superalloy was optimized for better mechanical properties to apply in turbine blades providing power.

It has been verified that appropriate Hf additions will improve plasticity of superalloys to a great extent [14]. In DD11 superalloy with 0.47 wt% Hf, creep rupture life has been upgraded from 320 h to 370 h under 1100 °C/130 MPa [15]. With a comprehensive first-principles study of solute elements in Ni-based binary alloys, it is defined that W have a relatively bigger radius and the lowest diffusion coefficient except Re, which has a strong affinity with creep [5].

Therefore, DZ951 superalloy is deliberately added with 1.5 wt% Hf and 3 wt% W named DZ951G superalloy. Previously, Cui [16] systematically studied the microstructure and deformation mechanism of the cast superalloy M951G that has the same composition as the directional solidified superalloy DZ951G. For DZ951G superalloy, it is also necessary to take the tensile test to provide the key information of the basic mechanical property.

During the tensile test, the main deformation mechanisms can be observed. One mechanism is dislocations shearing γ′ particles [17,18] and another is dislocations bypassing γ′ particles in which dislocation networks form at the interface of γ/γ′ [19,20]. From room temperature to intermediate temperature, anti-phase boundary (APB), stacking faults, dislocation reactions, and even the complex Kear–Wilsdorf lock can be observed [21,22,23,24]. At high temperatures, dislocation networks will generate with two modes.

In this work, the tensile property of DZ951G superalloy is investigated, and then the corresponding deformation mechanism with increasing temperature is analyzed in detail.

## 2. Experimental Procedure

The chemical composition of DZ951G superalloy is given in Table 1. The master alloy was initially melted in a vacuum induction furnace and cast into a polycrystalline ingot of about 5 kg. In order to get directional-solidification superalloy, the polycrystalline ingot was remelted and poured into a ceramic mold in the directional solidification furnace, where the temperature was 1550 °C and the withdrawal rate was 6 mm/min.

Then, as mentioned above, the incipient melting temperature was precisely detected by Differential Thermal Analysis (DTA). According to the result, the heat treatment was designed as solubilized at 1210 °C/4 h (air cooling), following aging at 1100 °C/4 h (air cooling) and 870 °C/4 h (air cooling).

Which were 5 mm in diameter with a gauge length of 25 mm. These standard samples were tested on an AG-250KNE mechanical test machine (Janpanse Shimadzu, Tokyo, Japan) at room temperature up to 1100 °C within a temperature fluctuation of 2 °C. Before it yielded, the strain rate was kept at 0.15 mm/min, while after that, the strain rate reached 3 mm/min. More than two parallel samples were tested at each temperature.

The as-cast, heat-treated and tensile ruptured samples were etched by chemical etching and electrolyzing. The corresponding chemical etching solution is composed of 20 g CuSO_4_ + 100 mL HCl + 5 mL H_2_SO_4_ + 80 mL H_2_O, and electrolyzing solution 300 mL H_3_PO_4_ + 20 mL H_2_SO_4_ + 30 g CrO_3_. The microstructure and phases were analyzed using the JMS-6301F field-emission scanning electron microscope (SEM, Thermo Fisher Scientific, Waltham, MA, USA).

After the tensile test, the thin foils were horizontally cut about 5 mm away from the fracture surfaces with a thickness of 500 µm, and subsequently ground down to 50 µm and punched. Then, after being perforated by a twin-jet electropolishing device in alcohol solution with 10 vol% perchloric acid at −25 °C and 20 V, these foils were prepared for transmission electron microcopy (TEM). Dislocation structure was investigated by a JEM (Japanese electronics, Tokyo, Japan) 2100 TEM at 200 kV.

## 3. Results

### 3.1. Microstructures Compared between As-Cast and Heat-Treated

**Figure 1:**
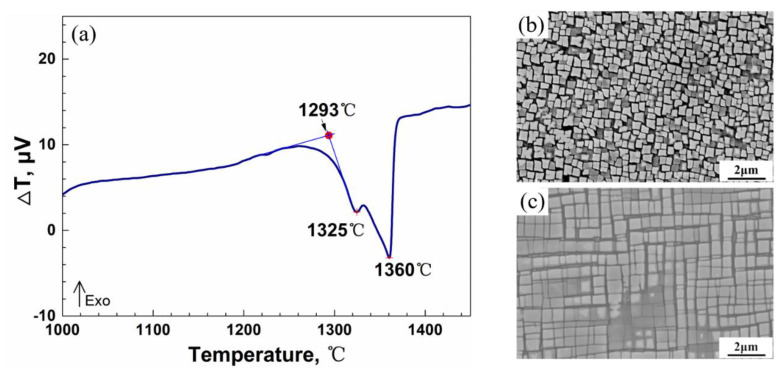
(**a**) DTA curves of DZ951G superalloy, (**b**) the ascast microstructures, and (**c**) the heat-treated microstructures.

In order to obtain its remarkable mechanical properties, a deliberate heat treatment regime was essential, formatively shaping γ′ particles and reducing eutectic volume fractions. Figure 1 displayed different phase-transition temperatures detected by Differential Thermal Analysis (DTA). It transpired that the incipient melting temperature of DZ951G superalloy was at 1293 °C, carbide melting at 1325 °C with γ matrix melting at a higher temperature of 1360 °C. Therefore, the heat treatment was designed as a solution at 1210 °C/4 h (air cooling), following aging at 1100 °C/4 h (air cooling) and 870 °C/4 h (air cooling).

After a standard process of being heat-treated, irregular γ′ particles were shaped into cubic particles, as compared with Figure 1b,c. Additionally, a great number of cubic tertiary γ′ particles could be observed in γ channels. As such, it is considered that the DZ951G superalloy has an excellent mechanical property in view of its ideal two-phase structures.

### 3.2. Tensile Behaviors

**Figure 2:**
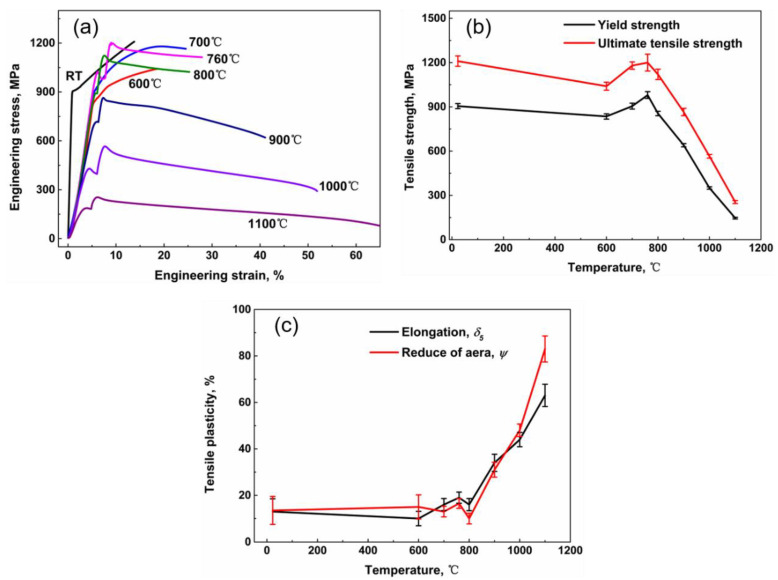
Tensile properties of DZ951G alloy. (**a**) Engineering stress–engineering strain curves; (**b**) strength with various temperatures; (**c**) plasticity with various temperatures.

The tensile properties of DZ951G superalloy under aviation standards are exhibited in Figure 2. According to the engineering stress–engineering strain curves of DZ951G superalloy, some essential information about the mechanical properties is shown. In Figure 2b, there is a characteristically abnormal yield point with a changed temperature which is common in materials containing L1_2_ structure, particularly in Ni-based superalloy [9]. Initially, the yield strength of DZ951G superalloy decreased slowly to 835 MPa from room temperature to 600 °C, then gradually increasing to the maximum 980 MPa at 760 °C. After that, there was a sharp drop to 640 MPa around 900 °C, which has a significant implication for its industrial application. Finally, the yield strength came to a dramatically low level of 146 MPa at 1100 °C. Accordingly, the ultimate tensile strength was maintained in accordance with the yield strength.

After reaching the yield strength at which dislocations were supposed to be operative, work-hardening behaviors appeared. Given the necking, it could be clearly observed that there was continuous work- hardening behavior from room temperature to 760 °C, but what was slightly abnormal was that work hardening at 760 °C was much stronger than that at 600 °C. Then, at a higher temperature above 900 °C, an equilibrium between work hardening and annealing with the almost horizontal stress line appeared.

The elongation *δ_5_* and the average reduction of area *ψ* of DZ951G superalloy are shown in Figure 2c. It was found that at an intermediate temperature, the average elongation kept a high value of over 10%. At 900 °C, the average elongation was almost two times that at 760 °C, indicating the fracture mechanism has been translating. Beyond 1000 °C, the average elongation reached a higher level. The trend of the reduction of area (R/A) is similar to that of the average elongation.

### 3.3. Fracture Analysis

**Figure 3:**
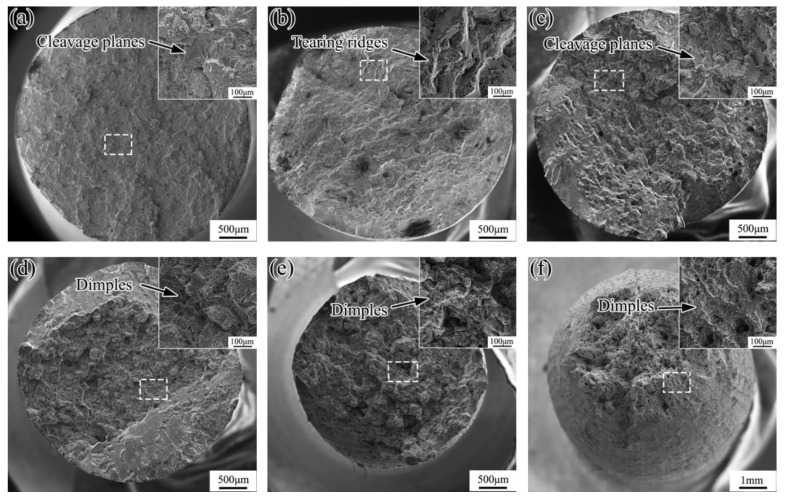
Fractographs of DZ951G alloy specimens at (**a**) room temperature, (**b**) 600 °C, (**c**) 760 °C, (**d**) 900 °C, (**e**) 1000 °C, and (**f**) 1100 °C.

Regarding tensile results of DZ951G alloy, six representative temperatures increased significantly to elucidate the distinction of fracture processes at room temperature, 600 °C, 760 °C, 900 °C and 1000 °C in Figure 3. From the macroscopic fractographs of room temperature, 600 °C, 760 °C, and 900 °C, there were obvious characteristics of transgranular fracture with lots of large sharp cleavage planes. However, it is noted that something still differed. In the corner of fracture, a great deal of small cleavage planes existed, proving brittle fracture at 760 °C in Figure 3c, coinciding with intermediate temperature brittleness in superalloys. However, dimples, as the feature of micropores aggregating and growing in ductile fracture, abounded there, and few cleavage planes can be seen at 900 °C in Figure 3d. This transition from brittle fracture into ductile fracture at 900 °C meant the deformation mechanism had been changed. Moreover, the feature of ductile fracture was entirely brought into ductile fracture at 1000 °C, in which primary large dimples were surrounded by secondary small dimples. At 1100 °C, the fracture stayed congruent with obvious necking, exhibiting higher elongation. In addition, brittle fracture at intermediate temperature shows the special brittleness of superalloys for their unique microstructures in Figure 3b–d [25].

### 3.4. Deformation Microstructures

#### 3.4.1. Low Temperatures

**Figure 4:**
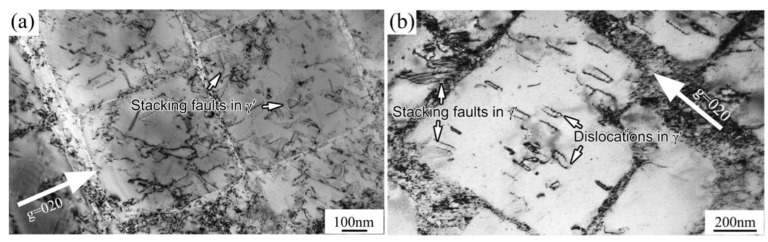
TEM images of deformation microstructures at low temperatures: (**a**) RT; (**b**) 600 °C.

When the applied stress exceeded the yield point of DZ951G alloy, a/2<101> matrix dislocations on the {111} slip planes in the γ channels began to generate, multiply, slip, cross-slip, and interact with each other, finally full in the channels under the integrative action of lattice mismatch and external force. A single a/2<101> dislocation cutting into γ′ particles can also be seen in Figure 4b; the straighter dislocation lines were, the higher energy dislocations had.

Meanwhile, both types of dislocation morphology can be observed in γ′ particles at room temperature in Figure 4a. One is isolated stacking faults, which was generally due to the mismatch of atom planes between two kinds of partial dislocations a/3<211> and a/6<121>, and another was a result of dislocations cutting through the γ′ particles [11,21].

#### 3.4.2. Intermediate Temperatures

At intermediate temperatures, there has always been a transitional phase of dislocation moving from shearing γ′ particles into by-passing, meaning climbing through γ′ particles for extra energy provided by heat with thermal diffusion motivation [16,17]. At 900 °C, this affect appeared more forcefully.

**Figure 5:**
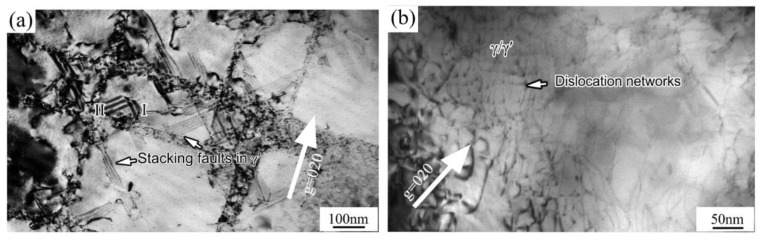
TEM images of deformation microstructures at intermediate temperatures: (**a**) 760 °C; (**b**) 900 °C.

**Figure 6:**
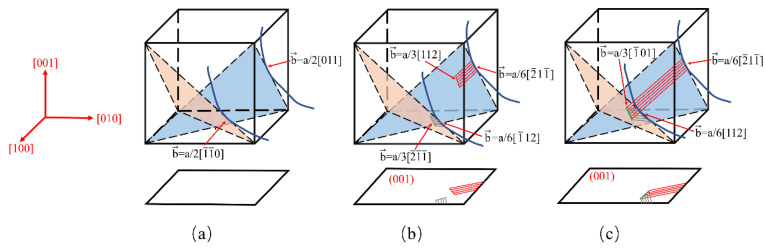
Schematic illustration of (**a**) dislocations moving to the surface of γ/γ′ phases, (**b**) the formation of stacking faults and (**c**) the reactions between stacking faults in the γ′ phase [16].

Stacking faults principally still appeared at 760 °C. However, it was curious that a few continuous stacking faults formed, crossing two γ′ particles displayed in Figure 5a. Meanwhile, dislocation reactions occurred between the stacking faults “I” and “II”, which would hamper other dislocations from moving [16]. The process of the formation of stacking faults and the reactions between stacking faults was vividly exhibited in Figure 6, which would be explained and discussed later.

At 900 °C, seldom or never could stacking faults be sought out and by heat with thermal diffusion motivation, dislocation bypassing was operated. Dislocation networks at the interface of γ/γ′ phases formed and are shown in Figure 5b.

#### 3.4.3. High Temperatures

**Figure 7:**
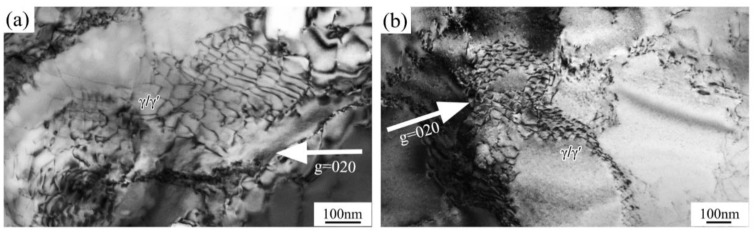
TEM images of deformation microstructures at high temperatures: (**a**) 1000 °C; (**b**) 1100 °C.

Above 900 °C, there was enough energy for dislocations motivated to bypass the γ′ particles, viz., the Orowan mechanism was operated [19]. During this process, dislocation networks at the surface of γ and γ′ phases formed, which have always played a crucial role in the creep property for restricting dislocations moving in γ channels. Two modes were identified: one was generated by a series of corrugated parallel dislocations meeting, reacting with dislocations in different slip planes, and another was brought out by cross dislocations cutting, leaving the dislocation nodes [26]. Both can be observed in Figure 7a,b. Dislocation networks also kept pace with this process, where dislocation networks at 1100 °C were slightly denser than those at 1000 °C.

## 4. Discussion

### 4.1. Stacking Fault

The stacking fault energy of pure Nickel was about 200mJ·m^−2^, while with some addition of elements such as W, Co, Re, Ru, stacking fault energy of Ni-based superalloys drastically dropped to the range from 20 to 30 mJ·m^−2^, and in some cases below 20 mJ·m^−2^ in γ matrix [22]. DZ951G alloy achieved this with the addition of 6.5 wt% of W and 5 wt% of Co. However, essentially in γ′ particles, it was that intrinsic stacking fault’s energy which was always approximately kept in 10 mJ·m^−2^. Meanwhile, the mismatched energy between γ and γ′ phases also promoted the formation of stacking faults, and with temperature increasing, the mismatched energy increases faster than the stacking fault energy, which was the primary reason stacking faults could be seen in γ′ rather than γ, as illustrated in Figure 4 and Figure 5.

Generally, a/2<101> dislocations primarily generated, multiplicated, and slipped in γ channels. It was inevitable that a/2<101> dislocations were destined to be impeded by γ′ particles, where optimal {111} slip planes disappeared for mismatch. Then, a/2<101> dislocations began to be partly dissociated into two partial dislocations a/3<211> and a/6<121>, where a/3<211> partial dislocations moved into γ′, causing a layer of atoms mismatching, and a/6<121> dislocations were still fixed at the interface of γ and γ′ [27], as shown in Figure 6b.

However, there were some stacking faults that did not make contact with the edge of γ′ particles in Figure 4a, which seemed unrealistic for the fixed a/6<121> dislocations at the interface of γ and γ′ phases. It was plausible that the dislocation configurations under TEM were only a matter of projection of authentic dislocations in space, where a/3<211> partial dislocations might cut from the bottom of cubic γ′ particles and cross the whole particle.

As for continuous stacking faults, Décamps considered that that the narrow γ channel, low stacking fault’s energy and enormous force in the matrix had a great influence on stacking faults continuing [28]. For DZ951G superalloy, there were narrow channels with lots of tertiary γ′ particles, and the stacking fault energy had been reduced by the high addition of Co and W. This was why, at 760 °C, continuous stacking faults could still be found during the tensile test, as shown in Figure 5a. 

Figure 6c precisely explains the reactions between the stacking faults “I” and “II”. Suppose there are two dislocations with different Burgers vectors a/2[011] and a/2[$\bar{1}\bar{1}$0], respectively, and when they sheared the γ′ particle, the splitting of dislocations occurred as follows [16].

(1) $$a/2[011]\;\to \;a/3[112]\;+\;a/6[\bar{2}1\bar{1}]\;+\;SF\;“I”$$

(2) $$a/2[\bar{1}\bar{1}0]\;\to \;a/3[\bar{2}\bar{1}\bar{1}]\;+\;a/6[1\bar{1}2]\;+\;SF\;“II”$$

Two a/3<112> dislocations sheared into γ′ particles, and a/6<121> dislocations were fixed at the interface of γ and γ′ phases. If a/3<112> dislocations on different slip planes kept moving, they would meet and react:

(3) $$a/3[112]+a/3[\bar{2}\bar{1}\bar{1}]\;\to \;a/3[\bar{1}01]$$

This process was reasonable given the geometric and thermodynamic conditions, leading to a fixed a/3<101> dislocation which could not slip on {111} planes [16]. The dislocation structure could hamper other dislocations from moving on {111} planes, to some extent, causing strong work hardening.

It had been reported that with the temperature increasing from 0 °C to 350 °C, stacking fault energy would be doubled in many fcc structure alloys. However, there was a common notion that the coefficients of thermal expansion in ordered, solid solutions were less than those in disordered solutions with temperature build-up, ultimately enhancing the effect of mismatch of γ and γ′ phases, which was beneficial for accelerating a/2<101> dislocation dissociating [29,30,31]. Therefore, the equilibrium appeared, which depended on the value of E_a/2<011>_ and E_a/6<211>_ + E_a/3<113>_ [16]. That was why stacking faults still could be seen in Figure 5a at 760 °C.

### 4.2. Relationship between Tensile Properties and Deformation Microstructures

**Figure 8:**
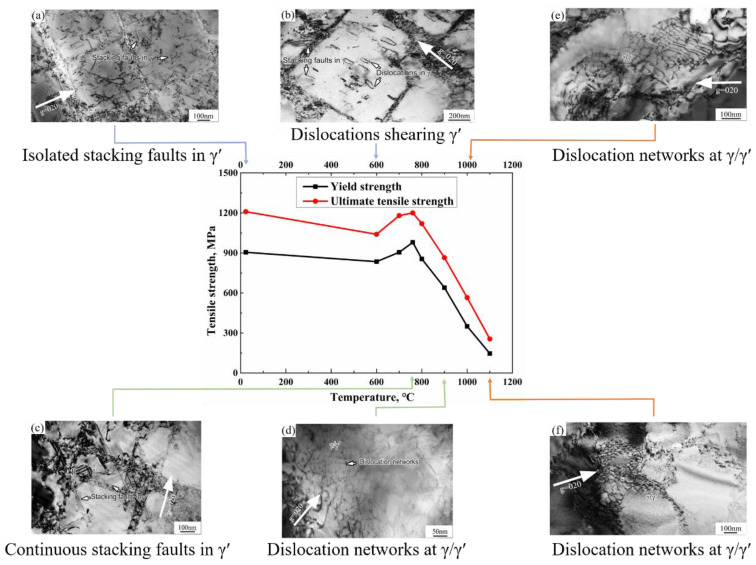
The tensile properties and corresponding deformation microstructures of M951G alloy at various temperatures. (**a**) RT; (**b**) 600 °C; (**c**) 760 °C; (**d**) 900 °C; (**e**) 1000 °C; (**f**) 1100 °C.

As demonstrated in Figure 8, tensile properties of DZ951G alloy had a strong dependence on temperature. Compared with DZ417G superalloy, DZ951G superalloy had a higher ultimate tensile strength where it was 1200 MPa at 760 °C. To some extent, yield strength kept a high numerical value below approximately 900 °C, and the maximum is 980 MPa at 760 °C, where a/2<101> dislocations shearing γ′ particles dominate in Figure 4 and Figure 5, while plasticity was at a low level for dislocations tangling and abounding in γ channels with severe strain localization. From room temperature to 900 °C, the brittle fracture with lots of large sharp cleavage planes dominated, but dimples, as the feature of the ductile fracture, can be seen at 900 °C in Figure 3d. This means the deformation mechanism had been changed, as shown in Figure 8.

Meanwhile, an abnormal yield point in superalloys has always existed, which is a characteristic phenomenon in alloys containing L1_2_ structure. One of the underlying reasons lay in the Kear–Wilsdorf lock, in which the slip planes of a/2<101> super screw dislocations in γ′ particles translated from {111} planes to the {100} planes [20]. Meanwhile, the reactions between stacking faults, forming the fixed dislocation structure in γ′ particles, also had a significant influence on the work hardening, as seen in Figure 5a. Moreover, the mismatch between γ and γ′ decreased by 0.5% from room temperature to 1000 °C [9]. This process enhanced the critical resolved shear stress on the {111} planes. This is why the abnormal yield strength 980 MPa existed at 760 °C in DZ951G superalloy. These indicated that mechanical properties were as a result of all kinds of dislocation competition.

At 900 °C, there was a sharp drop to 640 MPa due to the γ matrix being soft and grain boundaries failing easily at a high temperature. Above 1000 °C, the ductile fracture dominated with low strength and high elongation. Lots of dimples and obvious necking could be observed. Meanwhile, the deformation mechanism was transformed from shearing γ′ particles into bypass γ′ particles by thermal activation in Figure 8. When dislocations at different slip planes moved to the surface of γ and γ′ phases, it was difficult to shear into γ′ particles and dislocation networks began to form, containing corrugated parallel dislocations on different slip planes and dislocation nodes with cross dislocations reacting [32], as shown in Figure 7a,b. This was the original intention of designing superalloy that once dislocation networks were shaped, the only area that dislocations could move was the narrow γ channels under external force at high temperature, in which it was rather difficult for dislocations to move [9]. The denser the dislocation networks, the better the superalloy creep performance.

For the Orowan mechanism, the force obstructing dislocations moving in γ channels could be described as follows:

(4) $$\tau =\sqrt{\;\;\frac{2}{3}}\cdot \frac{Gb}{h}$$

In this formula, *G* is the shear modulus, and *b* and *h* are Poisson’s ratio and the width of γ channels, respectively. Suppose *G∙b* is a constant value and the width of γ channels at 1100 °C is two times hat at 1000 °C, the force at 1100 °C should be half of that at 1000 °C. Given γ′ phase dissolves at a higher temperature, this result was reasonable. In fact, the yield strength at 1000 °C and 1100 °C was 146 MPa and 350 MPa, respectively.

## 5. Conclusions

We conducted tensile tests of DZ951G superalloy at various temperatures, and corresponding microstructures and underlying deformation mechanisms were analyzed in detail. The following is a summary of the main findings:

1. The tensile strength of DZ951G superalloy was enhanced at intermediate temperature by the additions of Hf and W, in which the abnormal yield point 980 MPa existed at 760 °C;
2. In the present study, two types of stacking faults existed. One was isolated stacking faults below 600 °C, and another was continuous stacking faults at 760 °C. The reactions of stacking faults could also be observed;
3. The main deformation mechanism below 600 °C was dislocations shearing γ′ particles, but beyond 900 °C, bypassing γ′ particles dominated. At intermediate temperature, the transition from shearing to bypass appeared. The fracture translated from brittle fracture into ductile fracture at 900 °C.

## Figures and Tables

**Table 1:** Normal chemical composition of DZ951G superalloy.

Alloys	W	Hf	Nb	Cr	Co	Al	Mo	Others	Ni
DZ951G	6.5	1.5	2.2	9	5	6	3	0.091	Bal

## Data Availability

Data is contained within the article.

## References

[1] Xia W.S., Zhao X.B., Yue L., Zhang Z. (2020). A review of composition evolution in Ni-based single crystal superalloys. J. Mater. Sci. Technol..

[2] Perepezko J.H. (2009). The hotter the Engine, the better. Science.

[3] Tang Y.L., Huang M., Xiong J., Li J., Zhu J. (2017). Evolution of superdislocation structures during tertiary creep of a nickel-based single-crystal superalloy at high temperature and low stress. Acta Mater..

[4] Long H.B., Mao S.C., Liu Y.N., Zhang Z., Han X.D. (2017). Microstructural and compositional design of Ni-based single crystalline superalloys-A review. J. Alloys Compd..

[5] Varvenne C., Leyson G.P.M., Ghazisaeidi M., Curtin W.A. (2017). Solute strengthening in random alloys. Acta Mater..

[6] Hargather C.Z., Shang S.L., Liu Z.K. (2018). A comprehensive first-principles study of solute elements in dilute Ni alloys: Diffusion coefficients and their implications to tailor creep rate. Acta Mater..

[7] Arora K., Kishida K., Tanaka K., Inui H. (2018). Effects of lattice misfit on plastic deformation behavior of single-crystalline micropillars of Ni-based superalloys. Acta Mater..

[8] Zhang Y., Zuo T.T., Tang Z., Gao M.C., Dahmen K.A., Liaw P.K., Lu Z.P. (2014). Microstructures and properties of high-entropy alloys. Prog. Mater. Sci..

[9] Doi M. (1996). Elasticity effects on the microstructure of alloys containing coherent precipitates. Prog. Mater. Sci..

[10] Long H.B., Wei H., Liu Y.N., Mao S.C., Zhang J.X., Xiang S.S., Chen Y.H., Gui W.M., Li Q., Zhang Z. (2016). Effect of lattice misfit on the evolution of dislocation structure in Ni-based single crystal superalloys during thermal exposure. Acta Mater..

[11] Xia P.C., Yu J.J., Sun X.F., Guan H.R., Hu Z.Q. (2007). Influence thermal exposure on the γ′ precipitates and tensile property of DZ951 alloy. Mater. Charact..

[12] Xia P.C., Yu J.J., Sun X.F., Guan H.R., Hu Z.Q. (2007). Influence of thermal exposure on the microstructure and stress rupture property of DZ951 alloy. J. Alloys Compd..

[13] Chu Z.K., Yu J.J., Sun X.F., Guan H.R., Hu Z.Q. (2010). Tensile property and deformation behavior of a directionally solidified Ni-based superalloy. Mater. Sci. Eng. A.

[14] Zheng Y.R. (1986). Behavior of Hf in solidification of cast Ni-base superalloys. Acta Metall..

[15] Zhao Y.S., Zhang J., Luo Y.S., Li J., Tang D.Z. (2016). Effect of Hf and B on high temperature low stress creep behavior of a secondgeneration Ni-based single crystal superalloy DD11. Mater. Sci. Eng. A.

[16] Cui L.Q., Su H.H., Yu J.J., Liu J.J., Jin T., Sun X.F. (2017). Temperature dependence of tensile properties and deformation behaviors of nickel-base superalloy M951G. Mater. Sci. Eng. A.

[17] Zhang P., Yuan Y., Shen S.C., Li B., Zhu R.H., Yang G.X., Song X.L. (2017). Tensile deformation mechanisms at various temperatures in a new directionally solidified Ni-based superalloy. J. Alloys Compd..

[18] Tian C.G., Han G.M., Cui C.Y., Sun X.F. (2015). Effects of Co content on tensile properties and deformation behaviors of Ni-based disk superalloys at different temperatures. Mater. Des..

[19] Murakumo T., Kobayashi T., Koizumi Y., Harada H. (2004). Creep behaviour of Ni-base single-crystal superalloys with various γ′ volume fraction. Acta Mater..

[20] Qi D., Wang D., Du K., Qi Y., Lou L., Zhang J., Ye H. (2018). Creep deformation of a nickel-based single crystal superalloy under high stress at 1033 K. J. Alloys Compd..

[21] Zhang P., Yuan Y., Li J., Xu Y.F., Song X.L., Yang G.X. (2017). Tensile deformation mechanisms in a new directionally solidified Ni-based superalloy containing coarse γ′ precipitates at 650 °C. Mater. Sci. Eng. A.

[22] Ma S., Carroll L., Pollock T.M. (2007). Development of γ phase stacking faults during high temperature creep of Ru-containing single crystal superalloys. Acta Mater..

[23] Qi D.Q., Fu B.D., Du K., Yao T.T., Cui C.Y., Zhang J.X., Ye H.Q. (2016). Temperature effects on the transition from Lomer-Cottrell Locks to deformation twining in a Ni-Co-based superalloy. Scr. Mater..

[24] Kovarik L., Unocic R.R., Li P. (2009). Sarosi, C. Shen, Y. Wang, M.J. Mills, Microtwinning and other shear mechanisms at intermediate temperatures in Ni-based superalloys. Prog. Mater. Sci..

[25] Yang Y.H., Yu J.J., Sun X.F., Jin T., Guan H.R., Hu Z.Q. (2012). The influence of long-term thermal exposure on intermediate temperature brittleness behavior of a Nickel-base superalloy. Mater. Charact..

[26] Wang X.G., Liu J.L., Jin T., Sun X.F., Hu Z.Q., Do J.H., Choi B.G., Kim I.S., Jo C.Y. (2015). Dislocations motion during high-temperature low-stress creep in Ru-free and Ru-containing single-crystal superalloys. Mater. Des..

[27] Dang C.X., Zhang P., Li J., Gao Z.H., Li B., Gong X.F., Song X.L. (2020). The role of <112> {111} slip in the initial plastic deformation of Ni-base superalloys at room temperature. Mater. Charact..

[28] Décamps B., Raujol S., Coujou A., Pettinari-Sturmel F., Caron P. (2004). On the shearing mechanism of gamma’ precipitates by a single a/2〈110〉 dissociated matrix dislocations in Ni–based superalloys. Philos. Mag. A.

[29] Wang X.G., Liu J.L., Jin T., Sun X.F. (2014). The effect of ruthenium additions on tensile deformation mechanisms of single crystal superalloys at different temperatures. Mater. Des..

[30] Xiong X., Quan D., Dai P. (2015). Tensile behavior of nickel-base single-crystal superalloy DD6. Mate. Sci. Eng. A.

[31] Tian N., Tian S., Yan H. (2019). Deformation mechanisms and analysis of a single crystal nickel-based superalloy during tensile at room temperature. Mater. Sci. Eng. A.

[32] Agudo Jácome L., Nörtershäuser P., Somsen C., Dolouhý A., Eggeler G. (2014). On the nature of c0 phase cutting and its effect on high temperature and low stress creep anisotropy of Ni-base single crystal superalloys. Acta Mater..

